# Alterations in circulating immunoregulatory proteins discriminate poor CD4 T lymphocyte trajectories in people with HIV on suppressive antiretroviral therapy

**DOI:** 10.1128/mbio.02265-24

**Published:** 2024-09-17

**Authors:** Preeti Moar, Scott Bowler, Alan L. Landay, Sara Gianella, Lishomwa C. Ndhlovu, Thomas A. Premeaux

**Affiliations:** ^1^Division of Infectious Diseases, Department of Medicine, Weill Cornell Medicine, New York, New York, USA; 2 School of Medicine, University of Texas Medical Branch, Galveston, Texas, USA; 3 Division of Infectious Diseases and Global Public Health, Department of Medicine, University of California San Diego, San Diego, California, USA; University of California Davis, Davis, California, USA

**Keywords:** immune checkpoints, CD4 lymphocytes, immune reconstitution, composite biomarkers, HIV pathogenesis, machine learning

## Abstract

Despite the success in the management of HIV with antiretroviral therapy (ART), people with HIV (PWH) have a heightened state of immune activation and inflammation, and an estimated 10%–40% demonstrate poor CD4 T-cell reconstitution, thereby increasing their mortality and morbidity risk burden. Soluble immunoregulatory proteins that function in lymphocyte activation or inhibition are elevated in PWH and associate with T-cell dysfunction, HIV persistence, and are predictive of comorbid outcomes. Here, we measured a panel of 35 circulating immunoregulatory proteins in 116 PWH with variations in CD4 T-cell counts (poor CD4 trajectory: <200 cells/μl, *n* = 34 or immune competent: CD4 >500 cells/μl, *n* = 82) by Luminex. Participants were enrolled in the AIDS Clinical Trials Group Longitudinal Linked Randomized Trials cohort, had initiated ART on enrollment, and had been on suppressive ART for 1 year. Using non-parametric analysis, we found that the levels of CD276, ICOSL, BAFF, OX40, galectin-1, and galectin-9 were significantly higher in PWH with poor CD4 trajectories compared to individuals with immune-competent CD4 T-cell count. Notably, in logistic models, ICOSL and OX40 remained significant after adjusting for age and baseline plasma HIV RNA. Furthermore, Extreme Gradient Boosting machine learning models comprising co-stimulatory and inhibitory checkpoint proteins yielded high accuracy in classification of individuals with poor CD4 trajectories. In summary, we identified a novel signature of circulating immunoregulatory proteins indicative of poor CD4 trajectories that may serve as potential targets to monitor and manage immune perturbations more accurately in PWH during suppressive ART.

**IMPORTANCE:**

It is essential to track immune perturbations related to insufficient CD4 T-cell recovery in PWH on suppressive ART as those with incomplete reconstitution are at a greater risk of non-AIDS-related morbidity and mortality. Several inflammatory soluble mediators have associated with poor immune reconstitution and adverse morbid outcomes in PWH, yet their implementation into routine clinical care to guide management remains inconsistent. Circulating immune checkpoint proteins have been linked to dysregulated immune pathways during suppressive ART and may serve as improved surrogate markers of clinical relevance. Here we investigate soluble lymphocyte-associated immunoregulatory proteins in virally suppressed PWH with no reported co-morbid outcomes and varying CD4 T-cell counts, to reveal underlying pathways that remain perturbed despite ART. This novel signature of immunoregulatory markers pertaining to poor CD4 T-cell trajectories uncover previously overlooked immune checkpoints as important targets for clinical monitoring of PWH in the setting of durable viral suppression by ART.

## OBSERVATION

A hallmark of human immunodeficiency virus-1 (HIV) infection is the resultant depletion of CD4 T lymphocytes; however, with the advent of antiretroviral therapy (ART), a gradual recovery in CD4 T-cell counts occurs to an immune-competent range (>500 cells/μl) if viral suppression is retained. However, 10%–40% of people with HIV (PWH) maintain low CD4 T-cell counts (<200 cells/μl) despite successful virological suppression by ART (1). The etiology of immunological non-response can be multifactorial, including, but not limited to, age, host metabolic and genetic factors, coinfection, and longer duration of untreated HIV (2, 3). Importantly, PWH with incomplete reconstitution of CD4 T lymphocytes are at a greater risk of AIDS-related and non-AIDS-related morbidity and mortality. Furthermore, PWH with persistently low CD4 T-cell counts tend to have greater states of immune dysfunction, immune activation, and chronic inflammation (4–11). It is critical to identify functionally relevant biomarkers that define or predict poor CD4 T-cell trajectories in PWH as a proxy of treatment outcomes, disease progression, and concurrently inform on the mechanisms that yield an improvement in management.

Biomarkers of innate immune function and microbial translocation have been studied in the context of poor immune reconstitution in PWH (12), yet few studies have investigated the role of adaptive immune dysfunction in this population. Increased expression of immune checkpoint receptors on lymphocytes and their cognate ligands is associated with HIV disease progression (13–19). However, soluble forms of these immune checkpoints are detectable in the blood and may give a representation of lymphocyte activation and exhaustion status not only in the periphery but also in the functional capacity of cells residing in tissues. Soluble immune checkpoints predict disease outcomes in cancer and COVID-19 (20, 21). Soluble inhibitory immune checkpoints are highly associated with the frequency and functional capacity of HIV-specific T cells and persistent HIV reservoirs after ART implementation (22). Furthermore, many immune checkpoints have predictive value in the occurrence of morbid outcomes among PWH (23). However, the relationship between poor CD4 T-cell trajectories and lymphocyte-associated immunoregulatory proteins remains unclear. In the present study, we evaluated a panel of soluble co-stimulatory and inhibitory immune checkpoint proteins and explored relationships with CD4 T-cell trajectories in PWH with variable CD4 T-cell states, initiating ART without notable comorbidities.

## INDIVIDUAL AND COMPOSITIVE IMMUNE CHECKPOINTS DISCRIMINATE POOR CD4 T LYMPHOCYTE TRAJECTORIES

**Fig 1:**
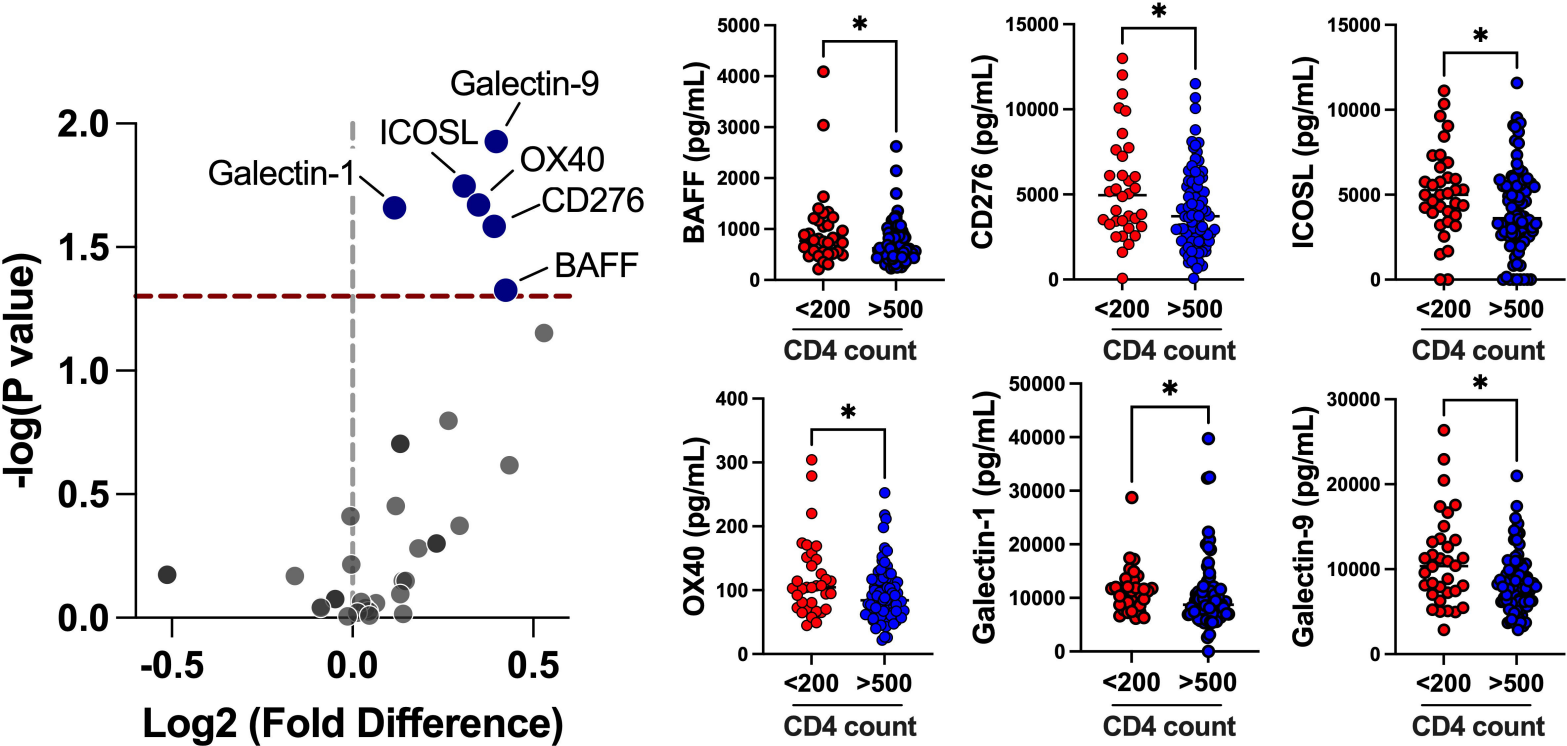
Elevated individual immune checkpoint proteins with poor CD4 T-cell trajectories. Volcano plots showing the estimated fold changes (*x*-axis) vs the -log_10_ *P*-values (*y*-axis) for each biomarker among poor CD4 T-cell count (<200 cells/μl) and immune-competent CD4 T-cell count (>500 cells/μl) groups. Distribution of immune checkpoints significantly elevated in individuals with poor CD4 T-cell trajectories. *P < 0.05.

We implemented an exploratory panel of 35 circulating co-stimulatory and inhibitory checkpoint proteins and their association with poor CD4 T-cell reconstitution among participants enrolled in the AIDS Clinical Trials Group Longitudinal Linked Randomized Trials cohort. Participants were stratified in poor CD4 T-cell count (CD4 <200 cells/μl, *n* = 34) or immune-competent CD4 T-cell count (CD4 >500 cells/μl, *n* = 82) subgroups and evaluated at 1 year post-ART during viral suppression (Table S1). Groups differed in age (*P* = 0.0060), baseline CD4 T-cell count (*P* < 0.0001), and baseline viral load (*P* < 0.0001), but no differences in sex, ethnicity, and parent study were observed. The differences observed in the CD4 trajectories may have been due to the difference in CD4 nadir and baseline viral load in the two subgroups. Furthermore, change in CD4 counts from baseline to year 1 post-ART significantly differed between the groups (*P* < 0.0001). Since the participants in the poor CD4 T-cell count subgroup had low CD4 counts to begin with, they had an overall improvement in CD4 counts due to initiation of ART; however, their rate of increase was significantly lower than those in the immune-competent CD4 T-cell count subgroup. Additionally, majority of participants had a CD4 count post-year 1 follow-up visit (median 796 days post-baseline); although the poor CD4 T-cell group increased significantly, all remained under a count of 500 cells/μl (Fig. S1). Among immunoregulatory proteins assessed, the poor CD4 T-cell trajectory group had significantly higher levels of the co-stimulatory immune checkpoints B cell-activating factor (BAFF; *P* = 0.0473), CD276 (B7-H3; *P* = 0.0325), inducible T-cell co-stimulatory ligand (ICOSL; *P* = 0.0203), and OX40 (*P* = 0.0213), compared to individuals with immune-competent CD4 T-cell counts (Fig. 1). Inhibitory immune checkpoint proteins galectin-1 (*P* = 0.0219) and galectin-9 (*P* = 0.0118) were also higher in individuals with poor CD4 T-cell trajectories (Fig. 1). ICOSL and OX40 remained significant in multivariate logistic regressions adjusted for age and baseline viral load (Table S2).

**Fig 2:**
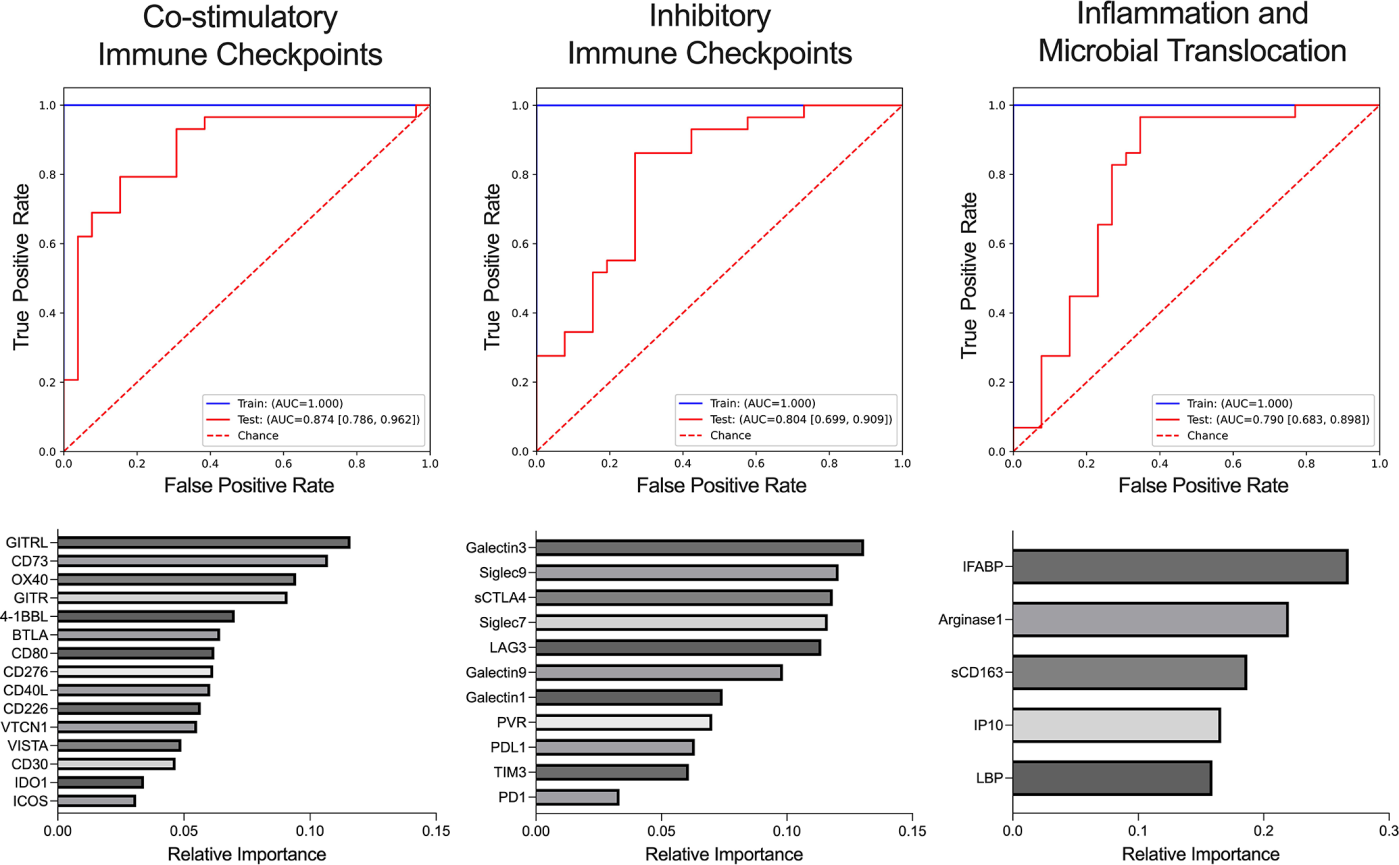
Compositive immune checkpoint pathways in the classification of CD4 T-cell trajectories. ROC curves illustrating XGBoost models classifying CD4 T-cell trajectories using co-stimulatory immune checkpoint proteins (AUC = 0.874), inhibitory immune checkpoints (AUC = 0.804), or soluble markers of inflammation and microbial translocation (AUC = 0.790). AUC-ROC measuring model accuracy for training and test sets are detailed in legend. XGBoost feature importance is determined by greatest *F*-score value, defined as the sum total of times a decision tree is split on a given feature.

As individual immune checkpoint proteins do not represent independent immunological states, we evaluated them in concerted stimulatory and inhibitory pathways (Table S3). Therefore, we next constructed Extreme Gradient Boosting (XGBoost) machine learning (ML) models to determine the classification of CD4 T-cell trajectories by immune activation or exhaustion composite markers. Areas under the curves (AUC) of the receiver operating characteristics (ROC) were calculated to assess the model performance, and recursive feature elimination was implemented to reduce model complexity by selecting optimized features (biomarkers), which carry significant and non-redundant predictive power to correctly classify participants. An XGBoost ML model comprising co-stimulatory immune checkpoint proteins (15 proteins selected out of 24 evaluated) yielded high accuracy in classification of individuals with poor CD4 T-cell counts (AUC-ROC = 0.874 [0.786, 0.962]; Fig. 2), while a model including inhibitory immune checkpoints (all 11 proteins selected) resulted in a slightly reduced performance (AUC-ROC = 0.804 [0.699, 0.909]; Fig. 2). Interestingly, these models yielded higher AUCs than an XGB model that included features of previously evaluated markers of inflammation and microbial translocation (5 proteins selected out of 12 evaluated; AUC-ROC = 0.790 [0.683, 0.898]; Fig. 2), further validating the importance of monitoring these pathways in PWH with poor CD4 T-cell outcomes. Model performance was comparable when all soluble markers (activation, inhibition, inflammation, and microbial translocation) were evaluated together (AUC-ROC = 0.817 [0.715, 0.919]; Fig. S2), suggesting the model did not benefit from expanding features beyond the co-stimulatory immune checkpoint proteins.

## CONCLUSION

In this study, we report that PWH with poor CD4 T-cell counts (>200 cells/μl) initiating ART and after a year of viral suppression demonstrate distinct soluble immune checkpoint signatures in blood compared to those presenting with immune-competent CD4 T-cell counts (>500 cells/μl). ML models consisting of immunoregulatory checkpoint proteins performed just as well as previously evaluated markers of inflammation and microbial translocation, in classifying PWH with divergent CD4 T-cell counts after 1 year of suppressive ART. More importantly, no measurable benefit was realized from expanding these features beyond the immunoregulatory checkpoint proteins. Our results provide new insight into immune checkpoints and their importance in monitoring CD4 T-cell outcomes and related pathways, particularly among those with poor immune reconstitution. Soluble forms of these immune checkpoint proteins are functionally active in immune regulation (20), and whether different levels in PWH contribute to discordant CD4 T-cell counts remains to be reconciled. As follow-up to our findings, using these soluble mediators to identify those at risk of poor CD4 T-cell recovery as well as investigating whether modulation of immune checkpoint pathways can reconstitute CD4 T-cell counts is worthy of further investigation.

Our study has some limitations. First, it is a cross-sectional evaluation of immune checkpoint proteins at 1-year post-ART initiation. The sample size is relatively small, especially in the poor CD4 trajectory group. However, results may be exploited into larger cohort study to assess whether they are linked to clinical outcomes in PWH lacking CD4+ T cell gain. Furthermore, a larger-scale longitudinal study following changes in immune checkpoints with CD4 T-cell trajectories will be essential to determine their predictive value. Second, women were underrepresented among participants. Since CD4 T-cell counts and VL differed between the two groups at baseline, immune checkpoint pathways could have been perturbed prior to ART initiation. Finally, members of the galectin and siglec families have complex roles in the inflammatory response (24, 25); thus, they can be driving multiple distinct pathways leading to inflammation and immune exhaustion. Nonetheless, we identified a novel signature of soluble lymphocyte-associated immunoregulatory proteins indicative of CD4 T-cell trajectories in PWH. Our observations may reveal potential targets for therapeutic intervention and new markers to enhance clinical monitoring of PWH on ART who have achieved virological suppression with incomplete immunological reconstitution.

## Data Availability

Individual participant data and a data dictionary defining each field in the set will be made available to investigators on a case-by-case basis via request to Advancing Clinical Therapeutics Globally for HIV/AIDS and Other Infections (ACTG). Completion of an ACTG Data Use Agreement may be required.

## SUPPLEMENTAL MATERIAL

Supplemental Material mbio.02265-24-s0001.pdf Supplemental methods and figures.

Table S1 mbio.02265-24-s0002.docx Participant demographics among poor and immune competent CD4 T-cell count groups.

Table S2 mbio.02265-24-s0003.docx Differences in biomarkers levels among poor and immune competent CD4 T-cell count groups.

Table S3 mbio.02265-24-s0004.docx Soluble markers classified by immune pathways.

ASM does not own the copyrights to Supplemental Material that may be linked to, or accessed through, an article. The authors have granted ASM a non-exclusive, world-wide license to publish the Supplemental Material files. Please contact the corresponding author directly for reuse.
