# Supplementary material for: Alterations in circulating immunoregulatory proteins discriminate poor CD4 T lymphocyte trajectories in people with HIV on suppressive antiretroviral therapy
Source: mBio. 2024 Sep 17;15(10):e02265-24. doi: 10.1128/mbio.02265-24 (PMC11481887; doi:10.1128/mbio.02265-24)
Supplement: Supplemental Material — Supplemental methods and figures. [file mbio.02265-24-s0001.pdf]

SUPPLEMENTAL MATERIAL

METHODS

Experimental Model and Study Participant Details.

NWCS 411 is a retrospective nested case-control study of PWH that were enrolled in the Advancing Clinical Therapeutics Globally for HIV/AIDS and Other Infections (ACTG) Longitudinal Linked Randomized Trials (ALLRT) cohort from 2001 to 2009. This study examined a multitude of potential predictive biomarkers of inflammation and immune activation and their relationships with adverse events and death (Tenorio et al. 2014). Participants included in our analysis did not experience a non-AIDS event/death during the ALLRT study in order to provide an estimate of the true association with CD4 count trajectories in the absence of non-AIDS events and death confounding variables.

All participants were ART-naive when enrolled in their original ACTG study and had plasma HIV RNA<400 copies/mL one-year post-ART initiation. Most study participants maintained their plasma HIV RNA<400 copies/mL after year 1; participants with values >400 copies/mL were included if preceding and subsequent HIV RNA values were <400 copies/mL without a change in ART regimen. By 48 weeks post-ART initiation, all participants were virally suppressed and the slope of decline in activation had stabilized (Gandhi et al. 2006). Participants (or, for minors, their parent or legal guardian) provided written informed consent, and institutional review board approval was obtained by each ACTG site. As part of their ACTG study, whole blood was obtained in tubes containing EDTA. Specimens were spun at 400 ×g for 10 minutes, and plasma was pipetted and spun again at 800 ×g for 10 minutes. Plasma was aliquoted, frozen, and stored at −70°C until assayed.

Biomarker Measurements. In this study, we measured the following biomarkers in available plasma samples using a custom multiplex immunoassay (Millipore): 4-1BB (CD137), 4-1BBL (TNFSF9), APRIL (TNFSF13a), BAFF (B cell activating factor; BLyS; TNFSF13b), CD276 (B7-H3), CD30 (TNFRSF8), CD40L (CD154), CD73 (5'-NT), DNAM-1 (DNAX accessory molecule; CD226), Galectin-1, Galectin-3, Galectin-9, ICOSL (inducible T cell co-stimulator ligand; B7-H2), IDO1 (Indoleamine-2,3-deoxygenase 1), OX40 (CD134), PVR (poliovirus receptor; CD155), Siglec-7 (sialic acid-binding Ig like lectin-7; CD328), Siglec-9 (CD329), VISTA (V-type immunoglobulin domain-containing suppressor of T cell activation; B7-H5), CD40, GITR (Glucocorticoid-induced tumor necrosis factor receptor-related protein), GITRL (GITR ligand), CD80, CD86, CD27, ICOS, CD28, HVEM (Herpesvirus entry mediator), BTLA (B and T lymphocyte attenuator), TIM-3 (T cell immunoglobulin mucin 3), LAG-3 (Lymphocyte-activation gene 3), PD-1 (Programmed death-1), PD-L1 (Programmed death ligand-1), CTLA-4 (Cytotoxic T-lymphocyte associated protein 4; CD152), and VTCN1 (V-set domain-containing T cell activation inhibitor 1; B7-H4). Data was acquired on a Luminex 200^{TM} analyzer and analyzed using MILLIPLEX® Analyst software (Millipore). All samples were analyzed in duplicates. Immunoregulatory protein results below the limit of quantification were set to an analytic lower limit. Analytic lower limits were taken as half of the largest lower limit for the specific protein. Any result that was below the identified largest lower limit was also set to the analytic lower limit.

Additional biomarker data were obtained from prior ACTG projects (ALLRT A5001, NWCS 329 and NWCS 387). Soluble plasma markers of inflammation and microbial translocation previously analyzed include the following: Arginase-1, IL-6 (Interleukin-6), TNFRI (Tumor necrosis factor

receptor I), TNFR-II, TLR2 (Toll-like receptor 2), CD163, CD14, suPAR (Soluble urokinase plasminogen activator receptor), IP-10 (Interferon gamma inducible protein 10), I-FABP (Intestinal fatty-acid binding protein), BDG (β-D-glucan), and LBP (Lipopolysaccharide-binding protein).

Statistics and Machine Learning Classification Modeling. Demographic and clinical characteristics are presented using the median (Q1, Q3) for continuous variables and frequency for categorical variables. Categorical variables were evaluated by Fisher's exact or Chi-square test. Individual biomarker differences were evaluated using Mann-Whitney test or adjusted multivariate logistic regression. CD4 T-cell count changes at baseline, year 1, and follow-up visit were evaluated by Mixed-effects analysis with Geisser-Greenhous correction and Tukey test for multiple comparisons.

Machine learning analyses was carried out in Python environment v3.8.8. Z-score standardization was applied to the dataset prior to employing machine learning algorithms. We implemented extreme gradient boosting (XGBoost) utilizing default settings and 10-fold cross validation. To improve efficiency and accuracy of our models, feature selection was employed to reduce model complexity by selecting optimized features (biomarkers) which carry significant and non-redundant predictive power to correctly classify participants. Feature selection was performed using Recursive Feature Elimination (RFE) with cross-validation (CV). RandomOverSampler (imblearn) was applied to account for class imbalance. Soluble biomarkers were divided into concerted stimulator and inhibitory pathways. Models trained on all features from each pathway to assess independent immunological states as classifiers of CD4 groups, retaining only features

with significant power to classify participants comprising the final model. We plotted the receiver operating characteristic (ROC) and calculated the area under the curve (AUC) to assess model performance. All AUC-ROCs are reported along with 95% confidence intervals. XGBoost feature importance is determined by greatest F-score value, defined as the sum total of times a decision tree is split on a given feature.

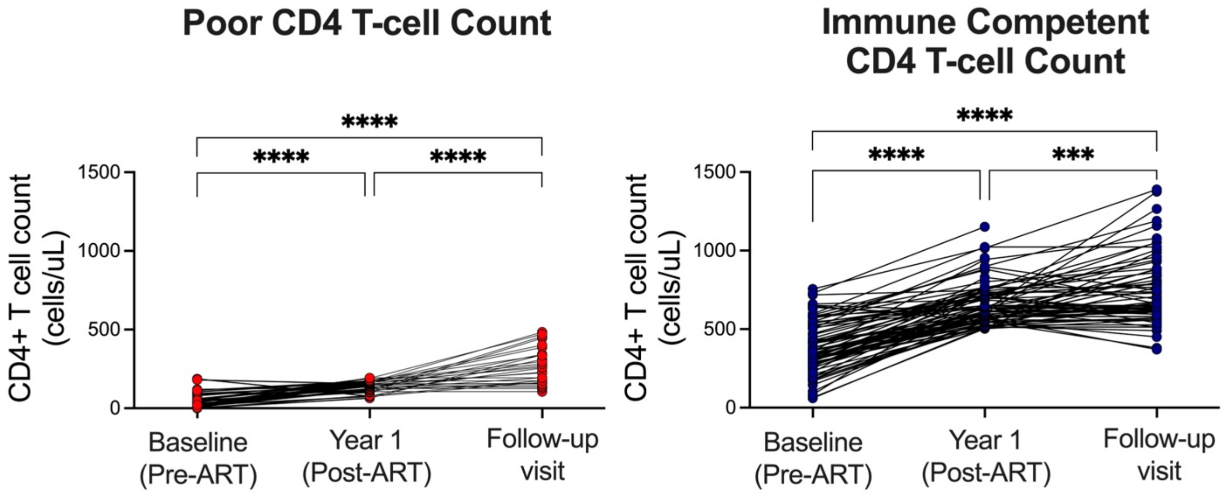

Figure S1. CD4 T-cell trajectories among participants. Changes in CD4 T-cell counts at baseline, one year after ART suppression, and follow-up visit (median 796 days post-baseline) among poor CD4 T-cell count and immune competent CD4 T-cell count groups. Differences were evaluated by Mixed-effects analysis with Geisser-Greenhous correction and Tukey test for multiple comparisons.

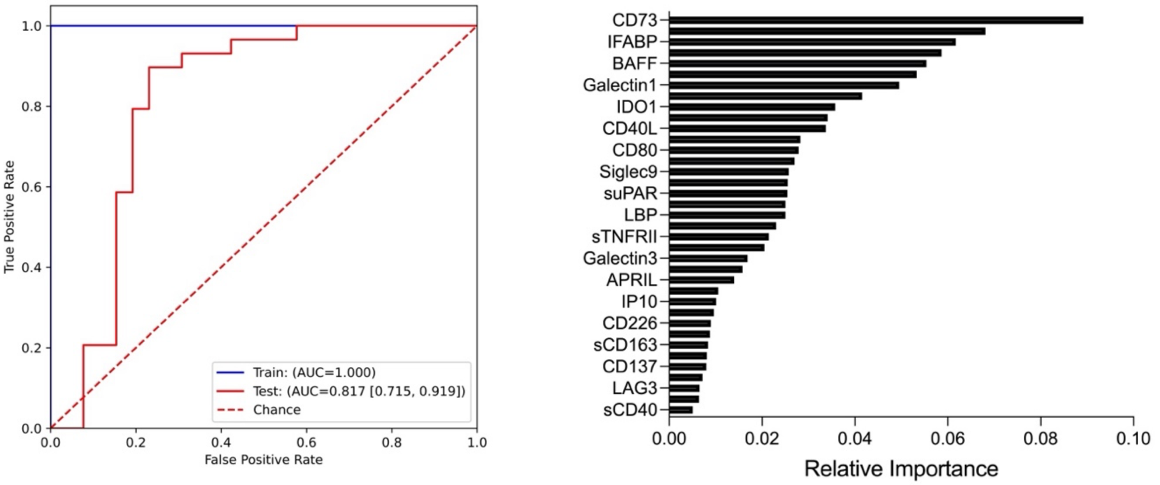

Figure S2. All immune checkpoint proteins in the classification of CD4 T-cell trajectories. ROC curves illustrating XGBoost models classifying CD4 T-cell trajectories using all checkpoint proteins (AUC=0.817). AUC-ROC measuring model accuracy for training and test sets are detailed in legend. XGBoost feature importance is determined by greatest F-score value, defined as the sum total of times a decision tree is split on a given feature.

References

Gandhi, Rajesh T., John Spritzler, Ellen Chan, David M. Asmuth, Benigno Rodriguez, Thomas C. Merigan, Martin S. Hirsch, et al. 2006. "Effect of Baseline- and Treatment-Related Factors on Immunologic Recovery after Initiation of Antiretroviral Therapy in HIV-1-Positive Subjects: Results from ACTG 384." *Journal of Acquired Immune Deficiency Syndromes (1999)* 42 (4): 426–34.

Tenorio, Allan R., Yu Zheng, Ronald J. Bosch, Supriya Krishnan, Benigno Rodriguez, Peter W. Hunt, Jill Plants, et al. 2014. "Soluble Markers of Inflammation and Coagulation but Not T-Cell Activation Predict Non-AIDS-Defining Morbid Events during Suppressive Antiretroviral Treatment." *The Journal of Infectious Diseases* 210 (8): 1248–59.
