# Supplementary material for: Alterations in circulating immunoregulatory proteins discriminate poor CD4 T lymphocyte trajectories in people with HIV on suppressive antiretroviral therapy
Source: mBio. 2024 Sep 17;15(10):e02265-24. doi: 10.1128/mbio.02265-24 (PMC11481887; doi:10.1128/mbio.02265-24)
Supplement: Table S1 — Participant demographics among poor and immune competent CD4 T-cell count groups. [file mbio.02265-24-s0002.docx]

| **Table S1. Participant demographics among poor and immune competent CD4 T-cell count groups** | | | | | |  |
| --- | --- | --- | --- | --- | --- | --- |
| **Characteristics** | | | **CD4 <200** | **CD4 >500** | **Total** | **P value** |
| **n** | | | 34 | 82 | 116 |  |
| **Age at parent study entry (years)** | | | 46.5 [41.75, 53.5] | 42.0 [36.75, 49.0] | 43.0 [38.0, 50.0] | 0.0077 |
| **Sex** | | |  |  |  | >0.9999 |
|  | Male | | 29 (85.3%) | 69 (84.15%) | 98 (84.5%) |  |
|  | Female | | 5 (14.7%) | 13 (15.85%) | 18 (15.5%) |  |
| **Race/Ethnicity** | | |  |  |  | 0.1214 |
|  | White Non-Hispanic | | 12 (52.2%) | 41 (47.2%) | 53 (45.7%) |  |
|  | Black Non-Hispanic | | 9 (35.8%) | 20 (27.3%) | 29 (25.0%) |  |
|  | Hispanic (regardless of race) | | 13 (11.2%) | 14 (21.7%) | 27 (23.3%) |  |
|  | Other | | 0 (0.0%) | 7 (3.7%) | 7 (6.0%) |  |
| **CD4+ T cell count (cells/μl)** | | | 146 [111.8, 166.0] | 637 [559.5, 743.3] | 575.0 [172.5, 709.5] | <0.0001 |
| **Baseline CD4+ T cell count (cells/μl)** | | | 35 [13.63, 80.88] | 365 [286.8, 521.3] | 300.5 [82.75, 426.9] | <0.0001 |
| **Change in CD4 count (baseline to year 1)** | | | 88.0 [-60.9, 119.4] | 295.9 [194.8, 376.8] | 234.9 [114.0, 331.0] | <0.0001 |
| **log10 HIV-1 RNA (copies/mL)** | | | 1.28 [1.11, 1.51] | 1.32 [1.11, 1.51] | 1.3 [1.11, 1.51] | - |
| **Baseline log10 HIV-1 RNA (copies/mL)** | | | 5.14 [4.57, 5.71] | 4.64 [4.35, 5.16] | 4.71 [4.39, 5.27] | 0.0016 |
| **ART regimens evaluated, by parent study** | | |  |  |  | 0.1091 |
|  | | ACTG 384:(AZT + 3TC vs d4T + ddI) + (EFV vs NFV vs NFV + EFV) | 12 (35.3%) | 17 (20.7%) | 29 (25.0%) |  |
|  | | ACTG 388: (AZT + 3TC vs d4T + 3TC) + (IDV vs NFV vs IDV + NFV) | 1 (2.9%) | 2 (2.4%) | 3 (2.6%) |  |
|  | | A5014: NVP + [LPV/r vs (ABC + 3TC + d4T)] | 0 (0.0%) | 1 (1.2%) | 1 (0.9%) |  |
|  | | A5095: AZT/3TC + (ABC vs EFV vs ABC + EFV) | 4 (11.8%) | 20 (24.4%) | 24 (20.7%) |  |
|  | | A5142: (EFV + AZT/d4T + 3TC) vs (LPV/r + AZT/d4T + 3TC) vs (EFV + LPV/r) | 13 (38.2%) | 21 (25.6%) | 34 (29.3%) |  |
|  | | A5202: (ABC/3TC vs TFV/FTC) + (ATV/r vs EFV) | 3 (8.8%) | 21 (25.6%) | 24 (20.7%) |  |
| Categorical variables are represented as frequency (%) and continuous variables as median [Q1, Q3] or median (range). P values are determined by Mann-Whitney for continuous variables and by Fisher’s exact test or Chi-square for categorical variables. Abbreviations: 3TC (lamivudine), ABC (abacavir), ATZ/r (ritonavir-boosted atazanavir), AZT (zidovudine), d4T (stavudine), ddl (didanosine), EFV (efavirenz), FTC (emtricitabine), IDV (indinavir), LPV/r (ritonavir-boosted lopinavir), NFV (nelfinavir), NVP (nevirapine), TFV (tenofovir) | | | | | | |
