# Supplementary material for: Alterations in circulating immunoregulatory proteins discriminate poor CD4 T lymphocyte trajectories in people with HIV on suppressive antiretroviral therapy
Source: mBio. 2024 Sep 17;15(10):e02265-24. doi: 10.1128/mbio.02265-24 (PMC11481887; doi:10.1128/mbio.02265-24)
Supplement: Table S2 — Differences in biomarkers levels among poor and immune competent CD4 T-cell count groups. [file mbio.02265-24-s0003.docx]

| **Table S2. Differences in biomarkers levels among poor and immune competent CD4 T-cell count groups** | | | | |  |
| --- | --- | --- | --- | --- | --- |
|  | Parameter | Poor CD4 count  (<200 cells/μl) | Immune competent CD4 count (>500 cells/μl) | p value | adjusted |
| Costimulatory Immune Checkpoints | APRIL | 372.35 (210.48, 475.39) | 373.83 (166.19, 448.58) | 0.3884 | - |
|  | BAFF | 972.37 (543.05, 1179.08) | 724.91 (448.50, 871.60) | **0.0473** | 0.0737 |
|  | BTLA | 4518.04 (1443.25, 7157.75) | 4381.68 (1241.25, 6754.50) | 0.9437 | - |
|  | CD137/4-1BB | 10.18 (4.55, 13.11) | 9.29 (4.90, 11.00) | 0.6561 | - |
|  | CD137L/4-1BBL | 37.90 (0.64, 18.19) | 54.08 (0.64, 29.02) | 0.6705 | - |
|  | CD27 | 1504.48 (805.92, 1867) | 1326.00 (688.81, 1661.00) | 0.5240 | - |
|  | CD276/B7-H3 | 5532.87 (3384.46, 7333.49) | 4215.42 (2271.18, 5807.52) | **0.0261** | 0.0528 |
|  | CD28 | 11617.81 (3485.75, 16752.75) | 12344.28 (3247.00, 17314.50) | 0.9015 | - |
|  | CD30 | 10.34 (0.12, 18.92) | 66.26 (0.12, 18.86) | 0.3935 | - |
|  | CD40 | 434.83 (255.43, 574.41) | 415.60 (287.32, 499.37) | 0.8729 | - |
|  | CD40L | 1487.83 (278.63, 2119.86) | 1661.60 (464.51, 2144.66) | 0.6783 | - |
|  | CD73 | 3116.36 (1598.61, 4010.07) | 2591.88 (1159.95, 3401.76) | 0.1592 | - |
|  | CD80 | 367.78 (132.28, 547.88) | 390.86 (144.65, 541.99) | 0.9111 | - |
|  | CD86 | 3390.02 (1491.25, 4754) | 3334.52 (1037.75, 4540.75) | 0.8634 | - |
|  | CD226/DNAM-1 | 3823.43 (91.52, 7663.86) | 2835.49 (91.52, 4904.68) | 0.1723 | - |
|  | GITR | 3292.85 (394.93, 2860.58) | 2681.35 (386.25, 2370.00) | 0.4234 | - |
|  | GITRL | 1222.45 (552.82, 1954) | 1263.50 (474.85, 1935.25) | 0.8398 | - |
|  | HVEM | 1177.21 (889.08, 1342.75) | 1083.23 (777.71, 1296.75) | 0.3528 | - |
|  | ICOS | 4557.43 (1769.5, 6719.5) | 4515.29 (1657.50, 6174.75) | 0.9531 | - |
|  | ICOSL | 5479.97 (4000.18, 6685.01) | 4424.82 (2848.71, 5602.60) | **0.0179** | **0.0087** |
|  | IDO1 | 163.00 (2.81, 104.02) | 148.67 (6.97, 148.94) | 0.1978 | - |
|  | OX40 | 118.11 (72.16, 148.92) | 92.75 (60.29, 115.45) | **0.0213** | **0.0357** |
|  | VISTA | 15.00 (0.01, 9.43) | 4.44 (0.01, 6.97) | 0.1470 | - |
|  | VTCN1 | 391.95 (56.62, 627.91) | 750.33 (6.45, 799.75) | 0.8395 | - |
| Inhibitory Immune Checkpoints | CTLA-4 | 324.73 (79.15, 448.06) | 294.56 (56.00, 416.87) | 0.9639 | - |
|  | PD1 | 3711.32 (1327.75, 4702.25) | 3621.04 (1216.75, 5049.25) | 0.9121 | - |
|  | PDL1 | 548.2 (182.43, 821.81) | 495.02 (149.08, 661.40) | 0.7105 | - |
|  | LAG3 | 83769.15 (41531, 114995) | 83988.39 (41446.50, 106777.50) | 0.6089 | - |
|  | TIM3 | 1704.58 (878.68, 1906.5) | 1548.20 (977.15, 1841.00) | 0.7095 | - |
|  | PVR | 55252.79 (37634.23, 63508.16) | 53471.10 (36410.35, 65546.49) | 0.9783 | - |
|  | Galectin-1 | 11346.79 (8550.81, 12649.8) | 10469.19 (7059.85, 11393.62) | **0.0219** | 0.2202 |
|  | Galectin-3 | 5592.65 (4000.79, 7050.45) | 5644.56 (4075.62, 6816.12) | 0.9880 | - |
|  | Galectin-9 | 10995.88 (7180.00, 13467.50) | 8345.00 (6182.50, 9985.00) | **0.0118** | 0.0665 |
|  | Siglec-7 | 5.14 (1.48, 3.98) | 3.80 (2.39, 4.27) | 0.2412 | - |
|  | Siglec-9 | 25.68 (8.81, 34.56) | 17.79 (7.58, 25.07) | 0.0703 | - |
|  | Biomarkers values (pg/mL) are presented as mean (Q1, Q3). Multivariate logistic models were adjusted for age and baseline viral load. | | | | |
