# Supplementary material for: Alterations in circulating immunoregulatory proteins discriminate poor CD4 T lymphocyte trajectories in people with HIV on suppressive antiretroviral therapy
Source: mBio. 2024 Sep 17;15(10):e02265-24. doi: 10.1128/mbio.02265-24 (PMC11481887; doi:10.1128/mbio.02265-24)
Supplement: Table S3 — Soluble markers classified by immune pathways. [file mbio.02265-24-s0004.docx]

| **Table S3. Soluble markers classified by immune pathways** | | |
| --- | --- | --- |
| **Co-stimulatory immune checkpoints** | | |
| 4-1BB/CD137 | CD30 | GITRL |
| 4-1BBL/CD137L | CD40 | HVEM |
| APRIL | CD40L/CD154 | ICOS |
| BAFF | CD73 | ICOSL |
| BTLA | CD80 | IDO1 |
| CD27 | CD86 | OX40 |
| CD276/B7-H3 | CD226/DNAM-1 | VISTA/B7-H5 |
| CD28 | GITR | VTCN1/B7-H4 |
| **Inhibitory immune checkpoints** | | |
| CTLA-4/CD152 | LAG-3 | Siglec-7 |
| Galectin-1 | PD-1 | Siglec-9 |
| Galectin-3 | PD-L1 | TIM-3 |
| Galectin-9 | PVR/CD155 |  |
| **Inflammation and microbial translocation** | | |
| Arginase-1 | Interleukin-6 | suPAR |
| BDG | IP-10 | TLR-2 |
| CD163 | LBP | TNFR-I |
| I-FABP | sCD14 | TNFR-II |
